# Purification of archetypal soybean root suberin mostly comprising alka(e)noic acids using an ionic liquid catalyst

**DOI:** 10.3389/fchem.2023.1165234

**Published:** 2023-08-10

**Authors:** Rita Escórcio, Armaan K. Sandhu, Artur Bento, Ana S. Tomé, Carlos J. S. Moreira, Volker S. Brözel, Cristina Silva Pereira

**Affiliations:** ^1^ Instituto de Tecnologia Química e Biológica António Xavier, Universidade Nova de Lisboa (ITQB NOVA), Oeiras, Portugal; ^2^ Department of Biology and Microbiology, South Dakota State University, Brookings, SD, United States; ^3^ Department of Biochemistry, Genetics and Microbiology, University of Pretoria, Pretoria, South Africa

**Keywords:** *Glycine max*, soybean root, purification of a suberin polymer, suberin *in planta*, quantification of polymeric features

## Abstract

Soybean (*Glycine max*) is an increasingly relevant crop due to its economic importance and also a model plant for the study of root symbiotic associations with nodule forming *rhizobia*. Plant polyesters mediate plant-microbe interactions with both pathogenic and beneficial microbes; suberin has been hypothesized to play a key role during the early steps of *rhizobia* attachment to the root. The downside is that suberin chemistry in soybean root is still scarcely studied. This study addresses this outstanding question by reporting a straightforward workflow for a speedy purification of suberin from soybean root and for its subsequent detailed chemical analysis. To purify suberin, cholinium hexanoate (an ionic liquid) was used as the catalyst. The ensuing suberin is highly esterified as observed by a precise Nuclear Magnetic Resonance quantification of each ester type, discriminating between primary and acylglycerol esters. Moreover, the composing hydrolysable monomers detected through GC-MS revealed that hexadecanoic acid is the most abundant monomer, similar to that reported before by others. Overall, this study highlights the adequacy of the ionic liquid catalyst for the isolation of suberin from soybean roots, where the polymer natural abundance is low, and builds new knowledge on the specificities of its chemistry; essential to better understand the biological roles of suberin in roots.

## Introduction

Soybean (Glycine max) is a model plant with high economical relevance used for animal feeding, biodiesel fuel production or bio-composite materials development (White et al., 2017). Moreover, it is a good source of dietary proteins with all essential amino acids and is a rich source of isoflavones (Messina, 2016). For all these reasons, the demand for this crop production is expected to double by 2050 (Ray et al., 2013). It is also an extremely important model for the study of root symbiotic associations with nodule forming rhizobia. Interaction between soil bacteria and plant roots is influenced by plant exudates and root surface properties (Albareda et al., 2006; White et al., 2017). In particular, root suberin is thought to influence the root microbiota and resistance towards root pathogens (Chen et al., 2022; Serra and Geldner, 2022). As root symbiotic associations with rhizobia occur in tissues enriched in lipid polyesters, especially suberin (Philippe et al., 2020; Serra and Geldner, 2022), a deeper understanding of their role in bacterial root attachment is crucial to decrease reliance on environmentally deleterious fertilizers while improving biological nitrogen fixation. In soybean, suberin is found in the epidermis within 5 mm from the root tip. This suggests that rhizobia with hydrophobic surface properties must attach to suberin as a prerequisite step leading to infection and nodule formation (Thomas et al., 2007; Ranathunge et al., 2008). This hypothesis furthered our interest in the suberin chemistry in soy roots that remains partially unresolved. Specifically, suberin is a complex multifunctional plant polyester deposited in the endodermis and epidermis of roots and outer barks of land plants (Harman-Ware et al., 2021; Woolfson et al., 2022). This polymer plays an essential role in the control of water loss, solute translocation, protection against pathogen invasion and response to wounding. Suberin chemical composition is species-specific and may change between plant organs and developmental stage. Typical monomers of the aliphatic suberin domain are long-chain and very-long-chain fatty acids, ω-hydroxy fatty acids, α, ω-dicarboxylic fatty acids, and primary fatty alcohols, which are linked through ester bonds (Serra and Geldner, 2022). The last decade was extremely prolific in generating advanced knowledge on the chemistry of the suberin polymer (Woolfson et al., 2022). One advance is the purification of suberin using an ionic liquid catalyst, which has been applied to extract suberin from the periderms of the outer bark of cork oak (Correia et al., 2020) and potato peel (Rodrigues et al., 2020). The catalyst (cholinium hexanoate) promotes a mild selective cleavage of the acylglycerol esters extant in the polymer, while preserving the primary aliphatic esters (Ferreira et al., 2013a). The ensuing suberin presents a highly esterified backbone, hence different from the mixture of the hydrolysable constituents that are recovered when harsh and/or non-selective hydrolyses are applied. Subsequent analyses of such esterified suberin, for example, through solution Nuclear Magnetic Resonance (NMR) lead to quantification of key molecular features, and through x-ray scattering to the disclosure of important structural insights (Bento et al., 2022). To date major advances on the characterization of suberin isolated using the ionic liquid catalyst focused on cork oak as a model, where suberin is particularly abundant (∼50%wt). One unresolved question is if the catalyst is biased towards a sub-type of suberin; *i.e.*, if the ionic liquid extracted suberin always illustrates the polymer present *in planta*, irrespectively of the extraction time used. Another unresolved question, is if the ionic liquid catalyst can be applied to purify suberin from plant roots where its abundance is extremely low. The present study builds foundational proof of concept that the ionic liquid catalysts is appropriate to ensure uncomplicated and efficient recovery of suberin present in soybean roots. The polymeric arrangement of the recovered root suberin (NMR), and its monomeric composition (GC-MS), were analyzed, and the data significance discussed.

## Materials and methods

### Chemicals

Sodium hydroxide (>98%) was purchased from José Manuel Gomes dos Santos; methanol (≥99.8%), dimethyl sulfoxide (DMSO, >99.99%), dichloromethane (>99.99%) and glycerol (≥99%) from Fisher Chemical; cholinium hydrogen carbonate (∼80% in water), hexanoic acid (>99.5%), hexadecane (>99%), 2.0 M (trimethylsilyl)diazomethane (TMSD) in hexane, N,O-bis(trimethylsilyl)trifluoroacetamide (99%) containing 1% (v/v) of trimethylchlorosilane (BSTFA + TMCS, 99:1), chromium (III) acetylacetonate (97%), N-hydroxy-5-norbornene-2,3-dicarboxylic acid imide (97%), 2-chloro-4,4,5,5-tetramethyl-1,3,2-dioxaphospholane (95%) and toluene (ACS >99.5%), heptadecanoic acid (≥98%), hexadecanedioic acid (96%) and pentadecanol (99%) from Sigma-Aldrich; hydrochloric acid (37%) from Riedel-de-Haën and deuterated dimethyl sulfoxide (DMSO-d6, >99.99%) from Merck. Hoagland’s basal salt mixture was acquired from MP Biomedicals. Cholinium hexanoate was synthesized by dropwise addition of hexanoic acid to aqueous cholinium hydrogen carbonate in equimolar quantities, as previously described (Petkovic et al., 2010).

### Plant material

Soybean (*Glycine max*) seeds of cv. Williams 82 were kindly provided by Dr. S Subramanian (South Dakota State University, SD, United States). For surface sterilization seeds were immersed in 2.25% sodium hypochlorite for 3 min, rinsed with sterile deionized water, and treated with 70% ethanol for 5 min. Seeds were washed seven times and soaked in sterile deionized water for 20 min before incubating on R2A agar at 30°C for 4 days to ensure sterility. Sterile seeds showing a radicle were potted in square pots (3″ x 3″ x 4” deep) containing a sterile mixture of 3:1 vermiculite: perlite. Pots were saturated with sterilized Hoagland solution (5 mM Ca(NO_3_)_2_.4H_2_O, 2 mM MgSO_4_.7H_2_O, 5 mM KNO_3_, 0.8 mM KH_2_PO_4_, 92 µM Na_2_Fe EDTA, 0.36 µM MnCl_2_, 0.034 µM ZnSO_4_, 1.15 µM H_3_BO_3_, 0.0125 µM CuSO_4_, and 52 µM H_2_MoO_4_), and incubated in a growth chamber at 25°C under lights for 14 h (day temperature) and 20°C for 10 h in the dark (night temperature). Plants were watered every 3 day and Hoagland solution was provided every seventh day. After 17 days, plants were harvested and the roots were decapitated, washed, dried at 50°C for 48 h, and autoclaved. Afterwards the roots were milled (Retsch ZM200 electric grinder; granulometry 0.5 mm; 10,000 rpm) and used for analysis.

Granulated cork (bark from *Quercus suber* L.) was obtained from Amorim & Irmãos SA (Santa Maria de Lamas, Portugal). Afterwards it was milled (Retsch ZM200 electric grinder; granulometry 0.5 mm; 10,000 rpm); then, the extractives were removed by sequential Soxhlet extraction with solvents of increasing polarity (dichloromethane, ethanol, and water) as previously described (Ferreira et al., 2012). The extractive-free preparations were washed in excess deionized water for complete removal of low-molecular-weight compounds and dried until constant weight at 60°C before use.

### Isolation of soybean roots suberin

Suberin was extracted from oak cork (hereafter simply defined as cork) or soybean roots, as previously described (Ferreira et al., 2012). In brief, 10 g of the plant source (previously ground to a fine powder) was mixed in 100 g of ionic liquid (cholinium hexanoate, synthesized as described above) and incubated for 2 h at 100°C with stirring. The reaction was stopped by the addition of 800 mL of DMSO. The insoluble residue was separated by filtration using a Nylon membrane filter (0.45 μm). Suberin materials were recovered from the filtrate through precipitation in excess water. The ensuing material, *i.e.*, purified suberin, was washed with an excess of deionized water with the aid of centrifugation (Beckman J2-MC centrifuge, 11655 *g* at 4°C for 30 min) and subsequently freeze-dried. The ensuing suberin powders were kept at room temperature until further analysis.

### Cryogenic grinding process

To analyze the *in planta* suberin profile, 100 mg of soybean roots were washed with 25 mL of deionized water, sonicated during 30 min, mixed and then centrifuged during 1 min, 2655 *g* at 4°C (Eppendorf centrifuge 5810 R) to recover the washed precipitate.

RESTCH Cryomill equipped with 5 mL grinding jars with two stainless steel grinding balls (4 mm) was used. Soybean roots (washed as described above) and isolated suberin were cryogenically milled at −196°C (liquid nitrogen) using 80 milling cycles as follows: 3 min of precooling followed by nine milling cycles, each cycle consisting of 3 min of milling at 30 Hz followed by 0.5 min of intermediate cooling at 5 Hz.

### Nuclear Magnetic Resonance (NMR)

NMR spectra of soybean roots (subjected to cryogenic milling as mentioned above) and suberin extracted from soybean roots with the ionic liquid were recorded using an Avance III 800 CRYO (Bruker Biospin, Rheinstetten, Germany). All NMR spectra (^1^H, ^1^H–^13^C HSQC, ^1^H–^13^C HMBC) were acquired in DMSO-*d*_6_ using 5 mm diameter NMR tubes at 60°C as follows: 15 mg of either soybean root or purified suberin in 500 μL of DMSO-*d*_6_ adding 10 µL of a solution of Benzene in DMSO-*d*_6_ as an internal standard. Quantitative ^31^P NMR spectra of suberin was also recorded using an Avance III 500 (Bruker Biospin, Rheinstetten, Germany) (Meng et al., 2019). MestReNova, Version 11.04-18998 (Mestrelab Research, S.L.) was used to process the raw data acquired in the Bruker spectrometers.

### Alkaline hydrolyses

To obtain suberin hydrolysates extracted from soybean roots or from cork with the ionic liquid catalyst (*i.e.*, composing of hydrolyzable monomers), as well as cork hydrolysates, the samples were submitted to alkaline hydrolysis. In brief, the samples were added to a solution of 0.5 M NaOH in methanol/water (1:1, v/v) at 95°C for 4 h (3:1 m/v). The mixture was cooled to room temperature and acidified to pH 3–3.5 with HCl 1 M, spiked with a known concentration of hexadecane (internal standard), and extracted three times with dichloromethane. Sodium sulphate anhydrous was added to the organic phase to remove water, which was then concentrated in a nitrogen flux. The dried combined organic extracts were immediately processed for GC-MS analyses (see below).

### Gas chromatography–mass spectrometry (GC–MS)

To quantify the amount of hydrolyzable monomers composing the purified suberin samples (or cork) an Agilent GC (7820A) equipped with an Agilent (5977B) MS (quadrupole) was used. For the first step of derivatization, 750 µL of MeOH:Toluene (2.5:1) and 500 µL of TMSD (2 M in hexane) were used (30 min, 90°C). For the second step of derivatization N,O-bis(trimethylsilyl)trifluoroacetamide containing 1% of trimethylchlorosilane in pyridine (5:1) were used (30 min, 90°C). The ensuing samples were analyzed by GC–MS (HP-5MS column) with the following ramp temperature: 80°C, 4°C/min until 310°C for 15 min. MS scan mode, with a source at 230°C and electron impact ionization (EI+, 70 eV), was used for all samples. The GC–MS was first calibrated with pure reference compounds (heptadecanoic acid, hexadecanedioic acid, pentadecanol and glycerol) relative to hexadecane (internal standard). Triplicates, each with technical triplicates were analyzed. Data acquisition was accomplished by MSD ChemStation (Agilent Technologies); compounds were identified based on the equipment spectral library (Wiley-NIST) and references relying on diagnostic ions distinctive of each derivative and its spectrum profile.

### Statistical analyses

Levene’s test was used to analyze the variance of the GC-MS data. Statistical differences for each monomer type between samples were analyzed using a two-way Analysis of Variance (ANOVA).

## Results

### The monomeric fingerprints of suberin samples sequentially purified from cork, using an ionic liquid catalyst, are similar

To specifically address if the cholinium hexanoate catalyst allows, after a defined reaction duration, recovery of a representative suberin polymer we resorted to cork (*i.e.*, outer bark of *Q. suber*) due to its high suberin content and extant knowledge on the chemistry of cork suberin purified using a similar process (Garcia et al., 2010; Ferreira et al., 2012). The ionic liquid catalyst mediates with time the cleavage of acylglycerol bounds, *i.e.*, the esterification of the purified suberin is time-dependent (Ferreira et al., 2012). Moreover, the 2 h reactions result in the extraction of only *ca.* 50% of the extant suberin in cork (Correia et al., 2020). Herein, suberin was sequentially extracted from cork, using the ionic liquid method (cholinium hexanoate, 2 h at 100°C with constant stirring) (Correia et al., 2020). After a first round of ionic liquid extraction, suberin was purified (precipitation in excess of deionized water), and the insoluble cork debris (washed and dried) were subjected to another round of suberin extraction with the ionic liquid. The hydrolysable monomers, resultant of an extended alkaline hydrolysis over each suberin sample, *i.e.*, first and second round of extraction, were quantified (µg·mg^−1^ of sample) through Gas Chromatography Mass Spectrometry (GC-MS), upon standard derivatization of free OH groups (Correia et al., 2020). The monomeric fingerprints of each hydrolyzable suberin were compared with that of the hydrolysable cork source - the monomeric fingerprint of suberin *in planta*. Results demonstrate, a similar diversity of hydrolysable monomers in the suberin samples from sub-sequent extractions by the ionic liquid catalyst, which are also similar to that of the non-extracted cork (Supplementary Table S1). This observation indicates that the ionic liquid method is unbiased to a specific sub-type of suberin. Moreover, suberin was also purified from *Pinus radiata* bark using the ionic liquid catalyst, where suberin abundance is very low (∼2% of the bark *wt*) (Bento et al., 2022). This result highlights that the reduction of suberin content in the raw material did not hindered the efficacy of the process. Hence, both observations suggest that the ionic liquid catalyst can allow recovery of suberin from soybean roots.

### A highly esterified soybean root suberin can be purified using the ionic liquid method

**FIGURE 1:**
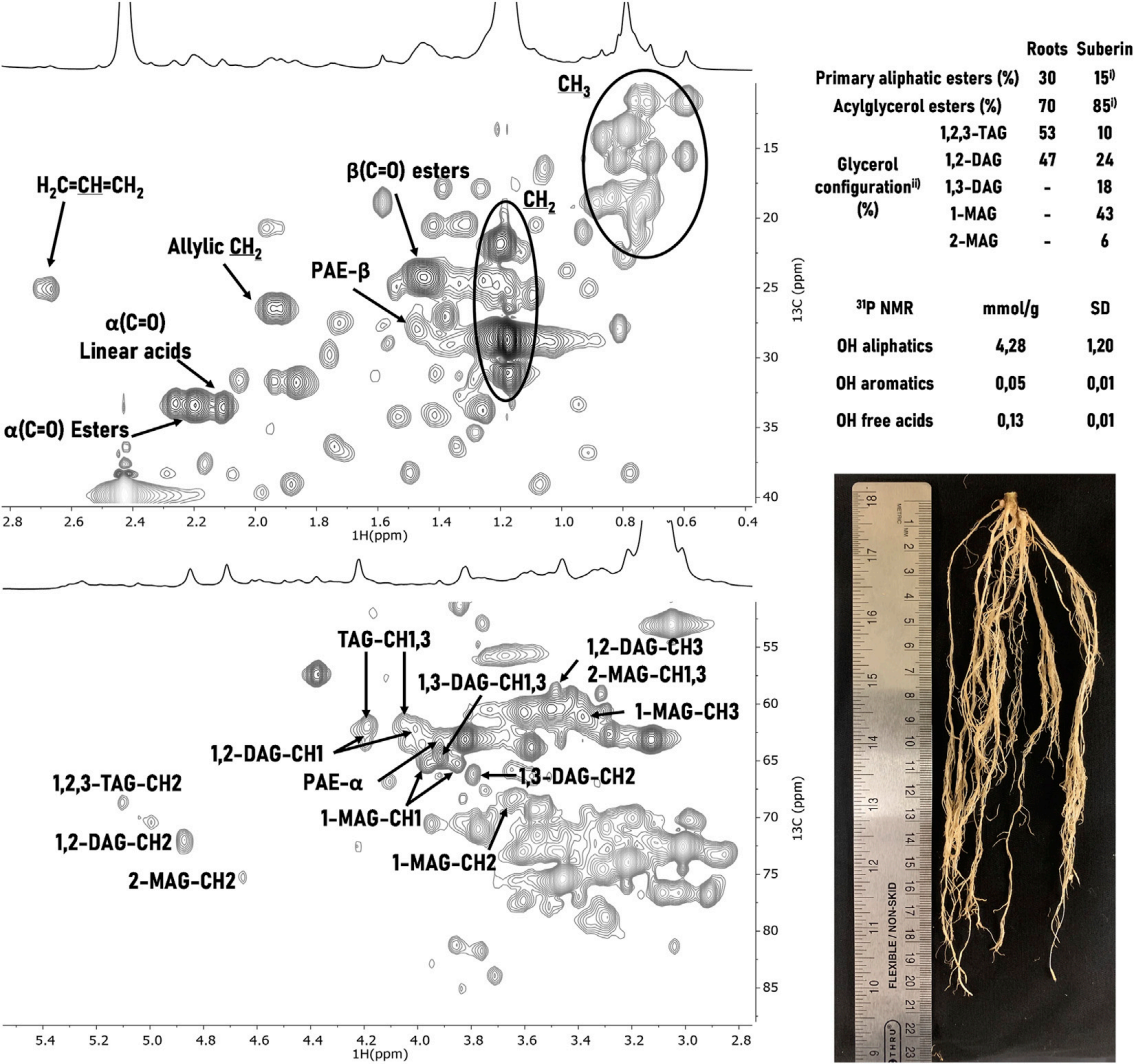
^1^H-^13^C HSQC NMR spectral characterization of soybean root suberin upon isolation using the ionic liquid extraction for 2 h. Some correlations (unlabeled) are uncertain or unidentified. The relative abundances of linear and acylglycerol esters 1) and of the of glycerol configurations 2) were inferred through the integration of the signals’ volume contour in the ^1^H-^13^C HSQC spectra of suberin *in planta* (roots) and of the extracted suberin (suberin). TAG, DAG, and MAG stand for triacylglycerol, diacylglycerol, and monoacylglycerol, respectively. The quantification of free acid and hydroxyl groups in the extracted suberin was determined through ^31^P NMR. A representative photo of the soybean roots is depicted.

To acquire further information on the chemical specificities of soybean suberin, 10 g of dried soybean roots were processed with cholinium hexanoate, following the abovementioned protocol. A total of 164 mg of suberin were recovered (1.64% of total root dry weight). The isolated suberin was analyzed through NMR: unidimensional proton (^1^H) (Supplementary Figure S1A) and two-dimensional Heteronuclear Single Quantum Coherence (HSQC) (Figure 1).

Through a combination of 2D spectra (^1^H−^13^C HSQC, and ^1^H−^13^C HMBC) and using as reference previous assignments of suberin (Correia et al., 2020; Bento et al., 2021; Bento et al., 2022), both CH_2_ and CH_3_ groups from aliphatic chains as well as alkene groups, most likely associated with 9,12-octadecadienoic acid (linoleic acid) and 9-octadecenoic acid (oleic acid), could be unequivocally assigned. Moreover, the signals related to α(C=O) acids at a^13^C shift of 33.13 ppm and ^1^H shift at 2.18 ppm, α(C=O) esters at a^13^C shift of 32.99 ppm and ^1^H shift at 2.27 ppm, and β-PAE (primary aliphatic ester) at a^13^C shift of 27.62 ppm and ^1^H shift at 1.54 ppm, were observed. In the glycerol CH-acyl region all five glycerol configurations were assigned: 1,2,3-triacylglycerol (TAG), 1,2-diacylglycerol (1,2-DAG), 1,3-diacylglycerol (1,3-DAG), 2-monoacylglycerol (2-MAG) and 1-monoacylglycerol (1-MAG). The relative contribution of each acylglycerol configuration was quantified (Bento et al., 2021) through the integration of each signal contour volume in the HSQC spectra (*insert* Figure 1). Moreover, the relative proportion of primary aliphatic ester *versus* acylglycerol esters was estimated. In addition, ^31^P NMR was used to quantify the amounts (mmol·g^-1^) of free acid and hydroxyl groups in the extracted suberin (*insert* Figure 1). This method, used extensively for the quantitative analysis of lignocellulosic polymers, involves the selective labelling of these groups (Meng et al., 2019). The amount of free hydroxyl groups (with only a minor contribution of OH aromatics) is much higher than that of free acid groups.

Finally, the NMR chemical signature of the ionic liquid isolated soybean root suberin was compared to that of the suberin found *in planta* (Supplementary Figure S1B). The last was directly solubilized in DMSO-*d*_6_ from soybean roots, which were subjected to cryogenic milling (80 cycles) (Correia et al., 2020), then washed with water and finally dried. The *in planta* suberin spectrum does not contain the 1,3-DAG, 1-MAG and 2-MAG signals; their presence in the ionic liquid extracted suberin denotes the catalytic activity of the ionic liquid that promoted a selective mild cleavage of some acylglycerol ester bonds.

**FIGURE 2:**
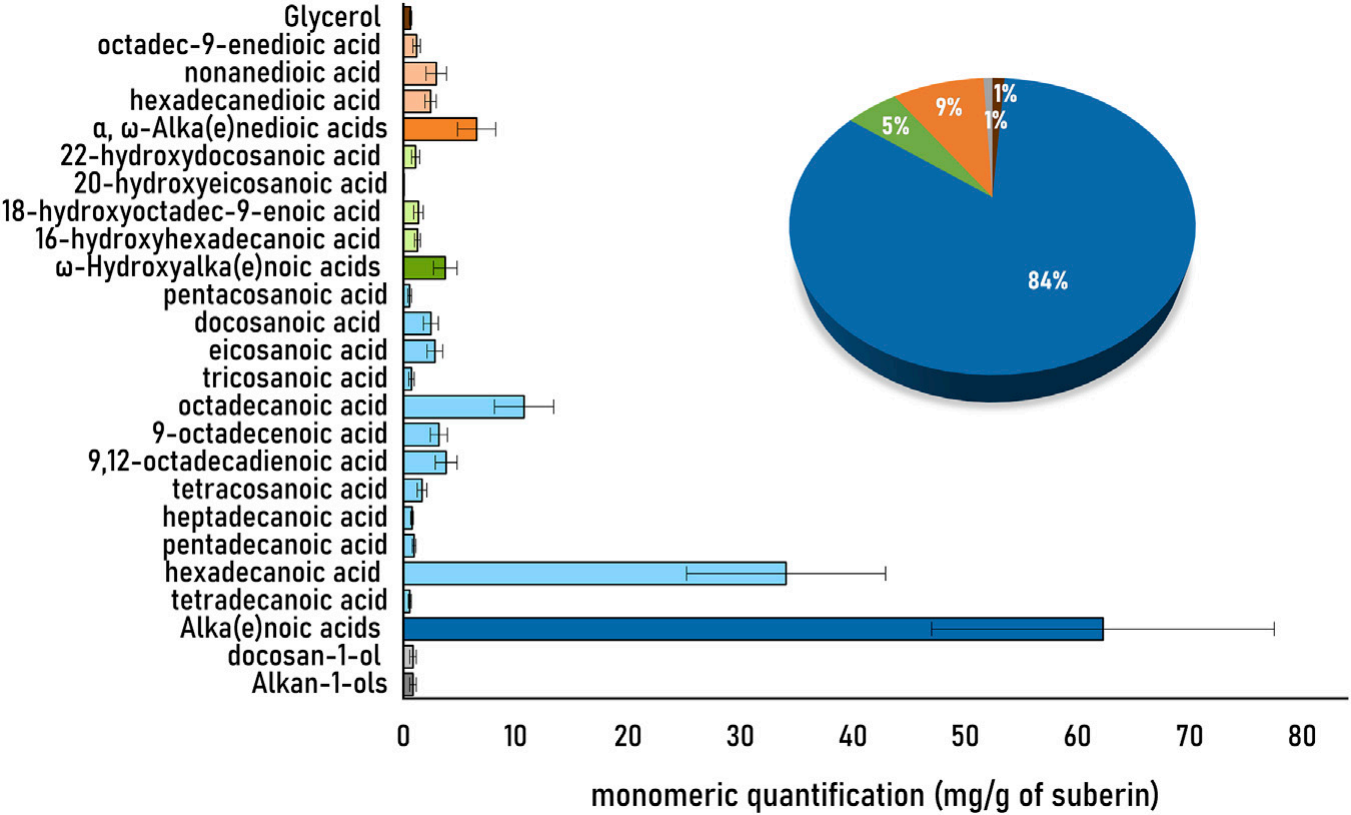
Quantitative analysis of the monomeric hydrolysates of the soybean root suberin extracted by the ionic liquid method. The ionic liquid extracted suberin was subjected to alkaline hydrolysis, and the hydrolysable monomers were quantified upon derivatization by GC-MS. Samples were analyzed in triplicate, with technical triplicates as well. The identified monomers are represented in the light colors, and their corresponding families by the dark colors. The pie graph represents the major families, namely, alka(e)oic acids (dark blue), α,ω-alka(e)nedioic acids (dark orange), ω-hidroxyalka(e)noic acids (dark green), alkan-1-ols (dark grey) and glycerol (brown).

The diversity of hydrolysable monomers detected in the soybean root suberin that was purified using the ionic liquid catalyst is depicted in Figure 2. The observed diversity of monomers is, in general, consistent to that reported before for suberin isolated from soybean roots using conventional extraction methods (Ghanati et al., 2005; Thomas et al., 2007), notwithstanding that root traits (including suberin composition) may differ in different cultivars (Figueroa-Bustos et al., 2020; Foxx and Kramer, 2021). Accordingly, the alka(e)noic acids (>80%) were the most abundant class of hydrolysable monomers detected, mostly hexadecanoic acid (it accounts for nearly 46% of the total monomeric content).

## Discussion

During suberin extraction from a plant source, the ionic liquid catalyst mediates with time the cleavage of acylglycerol bounds, *i.e.*, the glycerol content in the purified suberin is time-dependent. Accordingly, to purify suberin close to its native polymeric arrangement, short ionic liquid extraction periods are required. Taken cork as a model, the 2 h reaction results in the extraction of *ca.* 50% of the extant suberin but the native levels of glycerol are preserved in the polymer. The hypothesis that the monomeric composition of the extracted suberin is not affected by the usage of sequential reactions was validated here using cork as model. The results showed that the ionic liquid catalysis did not result in the purification of a specific sub-type of suberin when used to extract, consecutively, two times suberin from cork (Supplementary Table S1). This observation supports the adequacy of the ionic liquid to isolate a representative suberin polymer from its plant source. As such, together with our previous work on the isolation of suberin from *P. radiata* bark (Bento et al., 2022), where suberin is present at low abundance, similarly to that found in roots, suggested that the catalyst can be applied to recover suberin from soybean roots.

Analyses of the ionic liquid extracted soybean root suberin by NMR (Figure 1) showed a polymer enriched in alkene, methylene, methyl and acid groups (^1^H-^13^C NMR) as well as in OH aliphatic groups (quantitative ^31^P NMR data). Interesting, the *in planta* suberin shows high proportion of both TAG and 1,2 DAG (∼50% each); this feature is different from that observed in both cork (Correia et al., 2020) and *P. radiata* bark (Bento et al., 2022) where the observed ratio are 69:31 and 81:19, respectively, hence it is suggestive of an overall less cross-linked polymer. The ionic liquid purified soybean root suberin has 6-fold more acylglycerol esters (AGE) than primary aliphatic esters (PAE), hence showing a more pronounced cross-linked organization than linear. The PAE:AGE ratio is similar to that quantified in the *P. radiata* bark suberin (7-fold), but higher than that of cork suberin (2.3-fold). Finally, the overall distribution of the acylglycerol configurations is similar to that of *P. radiata* bark, both of which compared to cork suberin showed higher abundance of 1-MAG (Figure 1).

Finally, the root suberin showed a very low amount of OH aromatics (Figure 1, quantitative ^31^P NMR data) which could not be precisely assigned in the ^1^H NMR spectra due to the low signal intensity of these spectral region. The low abundance of aromatic features in the soybean root suberin–extracted suberin and *in planta* suberin–is likely explained by the specificity of the used cultivar. In fact, it has been reported that different cultivars have differences not only in response to drought and pathogens but also in root architecture and traits (Figueroa-Bustos et al., 2020; Foxx and Kramer, 2021). For example, the root epidermal wall compositions of cv. Conrad and cv. OX 760-6 soybean cultivars (resistant and susceptible to *Phytophthora sojae* respectively) differ greatly. The first is much richer in both the aliphatic and aromatic components of the polymer than the last (*p* < 0.05) (Fang, 2006). In addition, previous studies showed that the method is suitable to recover the aromatic constituents of suberin, which may undergo mild cleavage (<5%) under the catalytic conditions used (Bento et al., 2021). Taken as an example, the *P. radiata* bark suberin isolated using the ionic liquid catalyst showed the presence of aromatic features in the ^1^H NMR (*ca.* 2%, relative abundance), as well as the presence of the signal assigned to the esterified ferulic acid (trans-FA-α-ester) (Bento et al., 2022).

The soybean suberin monomeric composition as shown by GC-MS (Figure 2) revealed a suberin enriched in alka(e)noic acids (>80%). The observed profile is distinct from suberin samples extracted using the same ionic liquid method from the periderms of cork or potato peel (Rodrigues et al., 2020), yet it is comparable to that of *P. radiata* bark (Bento et al., 2022). The major distinctiveness is that both soybean root suberin and *P. radiata* bark suberin, are rich in alka(e)noic acids (nearly 60%, Figure 2) that are found at much lower levels in suberin from cork [12% (Correia et al., 2020)], white potato peel [6%, (Rodrigues et al., 2020)], and birch bark [9%, (Ferreira et al., 2013b)]. The observed suberin fingerprint suggests a unique polymeric arrangement in soybean root for the analyzed cultivar. The high abundance of alkanoic acids questions on the biological significance of such suberin chemistry. Further studies are needed to understand if the observed compositional features are systematically found in the root suberin of other soybean cultivars. Overall, the results emphasize the utility of the ionic liquid process to purify suberin from any plant source, irrespective of its native abundance. The recovered suberin is illustrative of the suberin occurring *in planta*. Despite low abundance of suberin in root, the ionic liquid extraction was time efficient and simple to implement. The recovered suberin largely preserves its acylglycerol-esterified backbone. Subsequent analyses through NMR and GC-MS allowed quantification of the suberin key molecular features and monomeric composition, respectively. Further methodological developments are possible to target suberin deposited in specific cell layers within the root. The methodological workflow here presented, which combines a catalyst that specifically mediates acylglycerol ester cleavage and quantitative solution NMR analyses, secures access to essential information on the polymeric arrangement of suberin. It is therefore undeniable that this workflow can help solving outstanding questions on suberin structural chemistry, biosynthesis and function in roots. Importantly, protocols described here to extract suberin will open the door to study the significance of suberin chemistry in the overall root microbiota and potential resistance to root pathogens, as well as the attachment of bacteria such as *rhizobia* to this polymer, as yet, poorly studied soybean root component.

## Data Availability

The original contributions presented in the study are included in the article/Supplementary Material, further inquiries can be directed to the corresponding author.
