# Supplementary material for: Purification of archetypal soybean root suberin mostly comprising alka(e)noic acids using an ionic liquid catalyst
Source: Front Chem. 2023 Aug 10;11:1165234. doi: 10.3389/fchem.2023.1165234 (PMC10448529; doi:10.3389/fchem.2023.1165234)
Supplement: Supplementary file 1 [file DataSheet2.PDF]

Table S1- Monomeric composition of the hydrolysates from cork and from cork suberin isolated using the ionic liquid method as determined by GC-MS. Sub_1 was obtained after a 2h extraction, and Sub_2 was obtained upon a serial 2h extraction over the insoluble leftovers of Sub_1 (see Materials and Methods). Statistically significant differences (*p*<0.05) in specific monomers are marked with *.

	Cork	Sub_1	Sub_2
Alkan-1-ols	2.45 ± 0.19	8.16 ± 1.01	4.86 ± 0.83
eicosan-1-ol	0.19 ± 0.06	0.71 ± 0.36	0.31 ± 0.07
docosan-1-ol	1.59 ± 0.19	5.62 ± 1.08	4.66 ± 0.92
tetracosan-1-ol	0.67 ± 0.1	2.00 ± 1.22	a
Alkanoic acids	5.29 ± 0.86	10.66 ± 0.49	10.65 ± 0.79
tetracosanoic acid	1.89 ± 0.32	2.47 ± 0.22	2.35 ± 0.19
hexadecanoic acid	0.57 ± 0.11	1.46 ± 0.09	1.58 ± 0.17
9,12-octadecadienoic acid	n.d.	0.2 ± 0.01	0.51 ± 0.29
9-octadecenoic acid	n.d.	0.71 ± 0.04	0.9 ± 0.18
octadecanoic acid	0.55 ± 0.08	1.16 ± 0.04	1.38 ± 0.23
docosanoic acid	2.29 ± 0.38	4.66 ± 0.53	4.02 ± 0.45
ω-Hydroxyalkanoic acids	142.23 ± 12.54	164.02 ± 28.08	198.62 ± 24.99
16-hydroxyhexadecanoic acid	2.51 ± 0.28	3.14 ± 0.04	3.55 ± 0.45
18-hydroxyoctadec-9-enoic acid	29.63 ± 3.35	34.87 ± 1.04	41.4 ± 6.69
18-hydroxyoctadecanoic acid	0.48 ± 0.04	n.d.	n.d.
20-hydroxyeicos-11-enoic acid	7.63 ± 0.43	7.26 ± 0.91	12.83 ± 4.94
20-hydroxyeicosanoic acid	3.12 ± 0.42	5.56 ± 0.82	5.01 ± 0.45
22-hydroxydocosanoic acid	43.26 ± 5.11	60.5 ± 3.36	59.91 ± 3.19
24-hydroxytetracosanoic acid	10.16 ± 1.04	15.36 ± 1.72*	13.3 ± 1.01*
?,18-dihydroxyoctadec-9-enoic acid	n.d.	5.70 ± 2.90	6.75 ± 1.85
?,?,?-trihydroxyoctadec-12-enoic acid	10.97 ± 0.72	2.32 ± 1.53	1.51 ± 1.32
9,10,18-trihydroxyoctadecanoic acid	10.27 ± 0.56	4.25 ± 0.71	2.86 ± 1.12
9,10-epoxy-18-hydroxyoctadecanoic acid	24.31 ± 2.2	38.91 ± 4.17	52.24 ± 10.15[a]
α, ω-Alkanedioic acids	61.09 ± 6.26	93.25 ± 8.01	82.2 ± 5.10
hexadecanedioic acid	5.84 ± 0.47	7.22 ± 0.32	7.29 ± 1.33
nonanedioic acid	0.81 ± 0.03	1.19 ± 0.11	1.43 ± 0.25
octadec-9-enedioic acid	11.69 ± 1.24	14.25 ± 0.59*	12.34 ± 1.02*
eicosanedioic acid	2.55 ± 0.35	4.71 ± 0.67*	4.39 ± 0.71*
9,10-dihydroxyoctadecanedioic acid	28.71 ± 3.06	46.88 ± 5.66	43.61 ± 3.64
docosanedioic acid	9.47 ± 1	16.25 ± 1.45	15.54 ± 0.6
9,10-dihydroxyeicosanedioic acid	2.01 ± 0.2	3.05 ± 1.01*	1.57 ± 1.00*
Glycerol (HPLC)	0.41 ± 0.42	63.78 ± 0.24	51.84 ± 0.22
Identification (%) – Total Area of the Chromatogram	68.89 ± 0.23	56.73 ± 14.93	66.32 ± 16.02
Identification (%) – Initial Mass	21.11 ± 1.98	28.46 ± 2.52	29.69 ± 2.14

a - Quantification impossible due to co-elution of different monomers.

n.d. – not detected

* Statistically significant difference between suberin samples (p<0.05)
