# Supplementary material for: Purification of archetypal soybean root suberin mostly comprising alka(e)noic acids using an ionic liquid catalyst
Source: Front Chem. 2023 Aug 10;11:1165234. doi: 10.3389/fchem.2023.1165234 (PMC10448529; doi:10.3389/fchem.2023.1165234)
Supplement: Supplementary file 2 [file DataSheet1.PDF]

a)

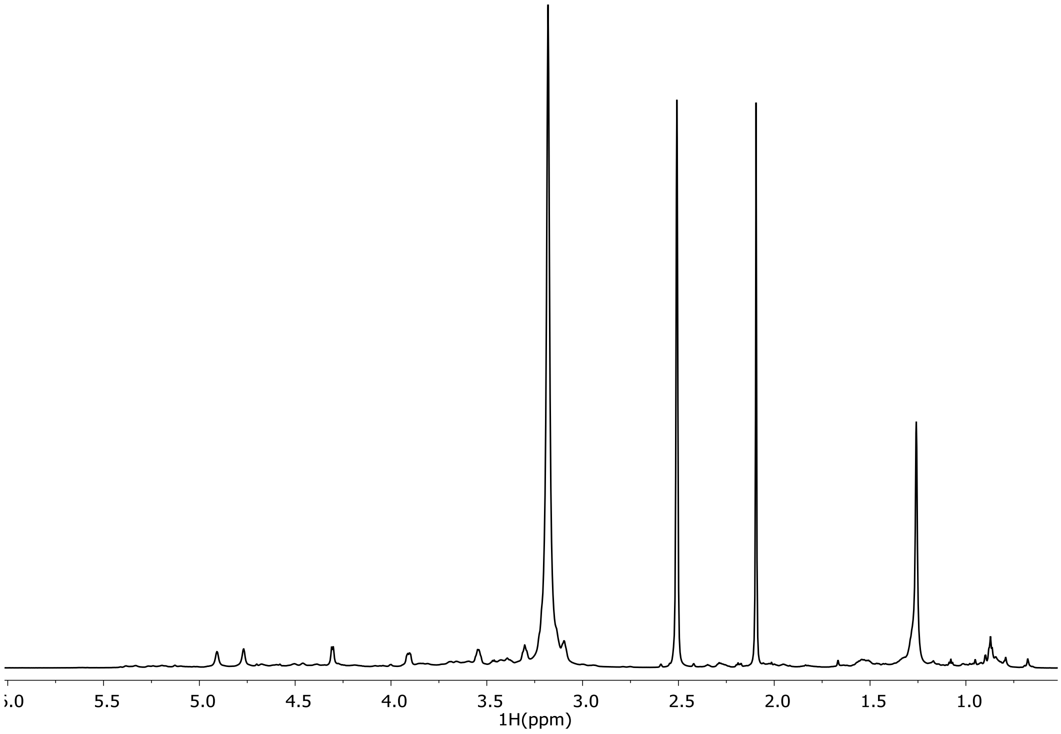

b)

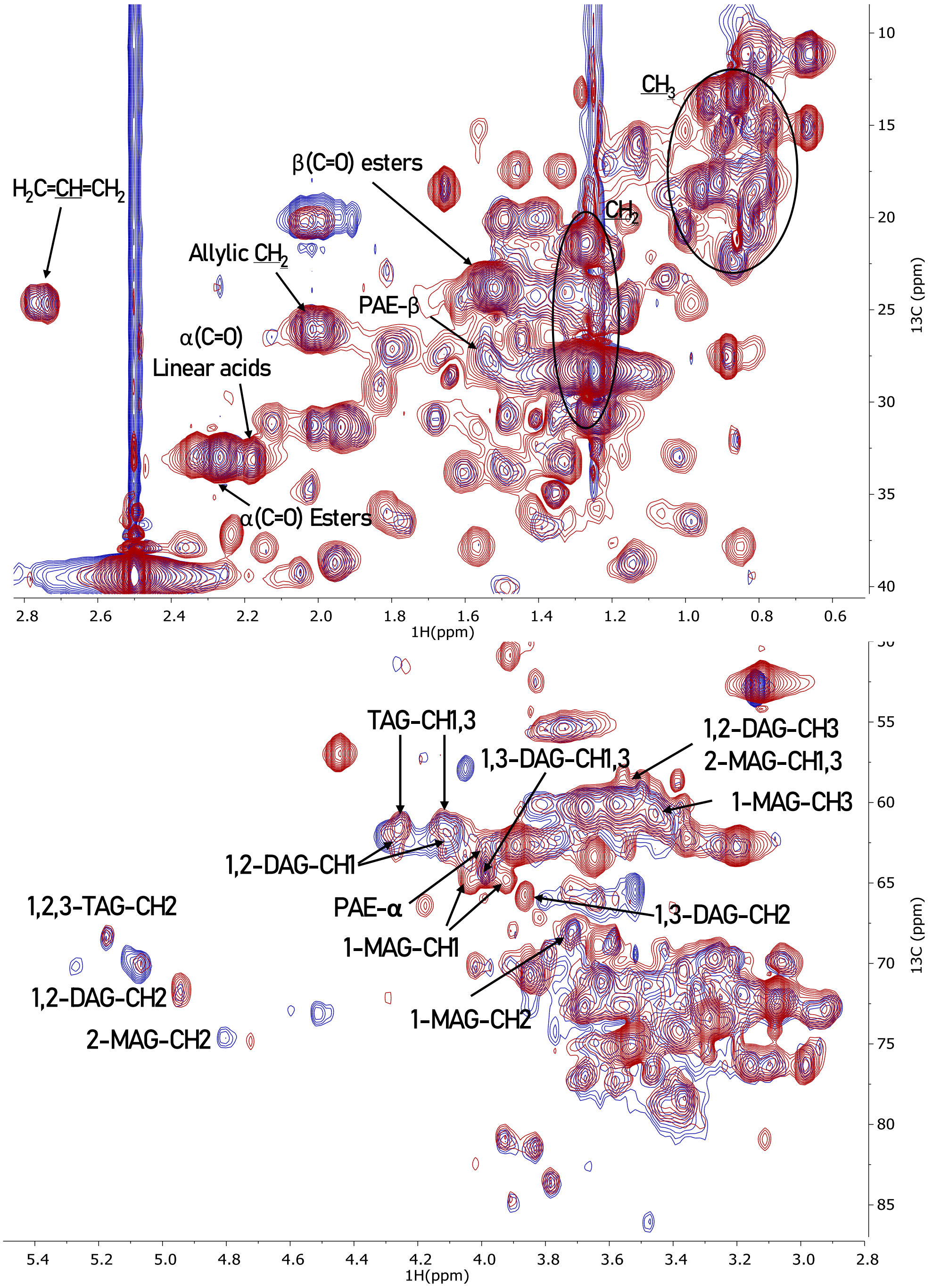

Figure S1- NMR spectral characterization. a) ^{1}H NMR spectrum of soybean roots suberin isolated using the ionic liquid process and b) ^{1}H-^{13}C HSQC NMR spectra of suberin purified from soybean roots using the ionic liquid extraction (red) and of suberin *in planta* (blue), *i.e.* directly solubilized upon extensive cryogenic milling of soybean roots. Some correlations (unlabeled) are uncertain or unidentified.
